# Duet of Death: Biventricular Thrombus in a Methamphetamine User

**DOI:** 10.7759/cureus.39917

**Published:** 2023-06-03

**Authors:** Jasninder Singh S Dhaliwal, Saad Ali Ansari, Sudeshna Ghosh, Akshit Chitkara, Umair Khizer

**Affiliations:** 1 Internal Medicine, University of California Riverside School of Medicine, Riverside, USA; 2 Internal Medicine, University of Texas Southwestern Medical Center, Dallas, USA

**Keywords:** thrombus, arterial emboli, emboli, substance induced disorders, substance, heart failure, heart, stroke, meth

## Abstract

We present the case of a 60-year-old male who developed an ischemic stroke due to left ventricular (LV) thrombus emboli as a complication of methamphetamine-induced cardiomyopathy. The patient had a history of methamphetamine abuse, hypertension, and ischemic stroke with no residual deficits presented with new onset slurred speech, left-sided weakness, and numbness for two hours. Computed tomography (CT) of the head showed no acute changes, and a tissue plasminogen activator was given in the emergency department within 30 minutes of arrival. Urine drug screen (UDS) was positive for methamphetamine, and magnetic resonance imaging (MRI) of the brain showed acute cortical infarcts in the right frontal lobe and parietal lobe and chronic infarct in the left occipital lobe. Transthoracic echocardiography showed bilateral ventricular thrombus and severely reduced ejection fraction of 20-25%. The patient had no evidence of thrombophilia and was started on a heparin drip for thrombus and goal-directed medical therapy for heart failure with reduced ejection fraction (HFrEF). Upon discharge, the patient was prescribed the oral anticoagulant rivaroxaban. The LV thrombus emboli were attributed to causing ischemic stroke. This case highlights the potential risk of ischemic stroke due to LV thrombus emboli in patients with methamphetamine-induced cardiomyopathy.

## Introduction

Methamphetamine use has become a major public health problem; it is a commonly available and inexpensive drug on the streets of America. This drug is a sympathomimetic amine; one of its remarkable and life-threatening effects is heart failure, which has been implicated as the second leading cause of death among methamphetamine users [1]. One of the devastating complications is thromboembolic events. Cardiac thrombus can form in various clinical conditions, frequently in myocardial infarction (MI) and cardiomyopathy. Heart failure with reduced ejection fraction (HFrEF) has long been known to cause the development of left ventricle (LV) thrombus. The systolic dysfunction likely increases the levels of prothrombotic cytokines and caters to a highly thrombogenic environment by creating long periods of blood stasis. Meurin et al. showed the risk of thrombus to be as high as 26%. Biventricular thrombi have also been reported but are relatively rare, even in patients with severe heart failure [2]. The optimal treatment for such a complicated condition remains very unclear. Therefore, we present a case report focusing on diagnostic imaging, therapeutic challenges, and further management of biventricular thrombi.

## Case presentation

This patient was a 60-year-old male with a six-month history of methamphetamine abuse, hypertension and previous ischemic stroke without residual deficits about six months ago. He had no family history of cardiovascular disease. He presented to the emergency department (ED) with acute onset of slurred speech, left-sided weakness, and numbness that started 2 hours ago. He also reported chest pain, palpitations, dyspnea, and diaphoresis. Code stroke was activated, and neurology was consulted in ED. On examination, he was alert but confused. His blood pressure was 154/122 mmHg, pulse was 98 beats per minute and regularly, respiratory rate was 24 breaths per minute, temperature was 36.6°C, and oxygen saturation was 95% on room air. The patient's neuro examination revealed several notable findings. He was alert but confused, with slurred speech. Additionally, he exhibited left-sided facial droop, and weakness in the left-sided arm and leg, with drift but the patient was able to move against gravity to some extent. Reduced sensations were noted on the left side of the body. Furthermore, the patient demonstrated limited coordination due to weakness in the left leg. The calculated NIHSS (National Institutes of Health Stroke Scale) score was 11. His cardiac auscultation revealed tachycardia with an S3 gallop and no murmurs or rubs. His chest was clear to auscultation bilaterally. His abdomen was soft and non-tender, with no organomegaly or masses. His peripheral pulses were weak and thready with no edema.

**Figure 1:**
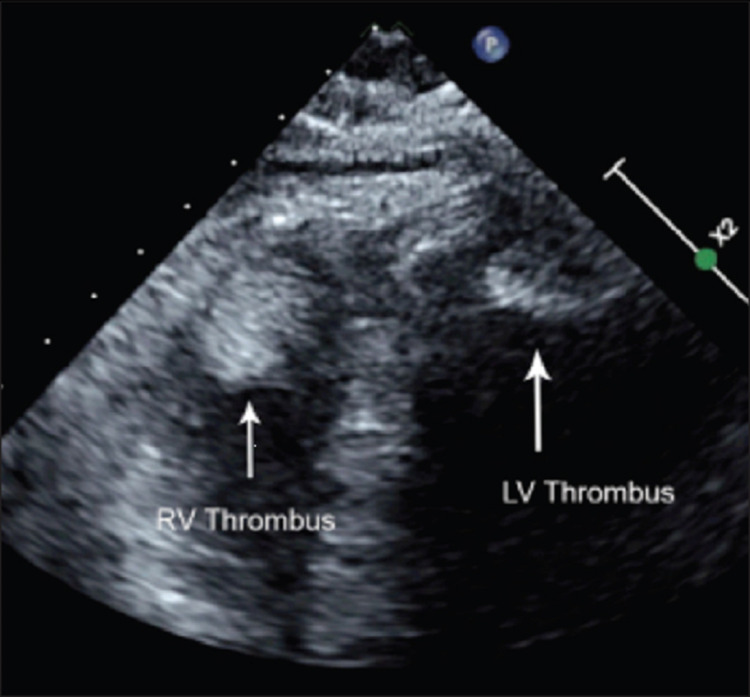
Echocardiogram shows the thrombus visible in right and left ventricles (white arrow) LV: left ventricular; RV: right ventricular

A baseline laboratory work-up showed minimally elevated creatinine and cardiac markers (troponin and creatine kinase). The urine drug screen (UDS) was positive for methamphetamine. His computed tomography (CT) head, electrocardiogram (ECG), and chest X-ray were unremarkable. The provisional diagnosis was an ischemic stroke and type-II MI. This patient received a tissue plasminogen activator (tPA) within 30 minutes of arrival. His magnetic resonance imaging (MRI) brain showed acute cortical infarcts in the right frontal lobe and parietal lobe, as well as chronic infarct in the left occipital lobe. The computed tomography angiography (CTA) of the head and neck showed no occlusive arteries. His echocardiogram (ECHO) showed a reduced ejection fraction of 20-25% with RV and LV apical thrombi (Figure 1).

**Figure 2:**
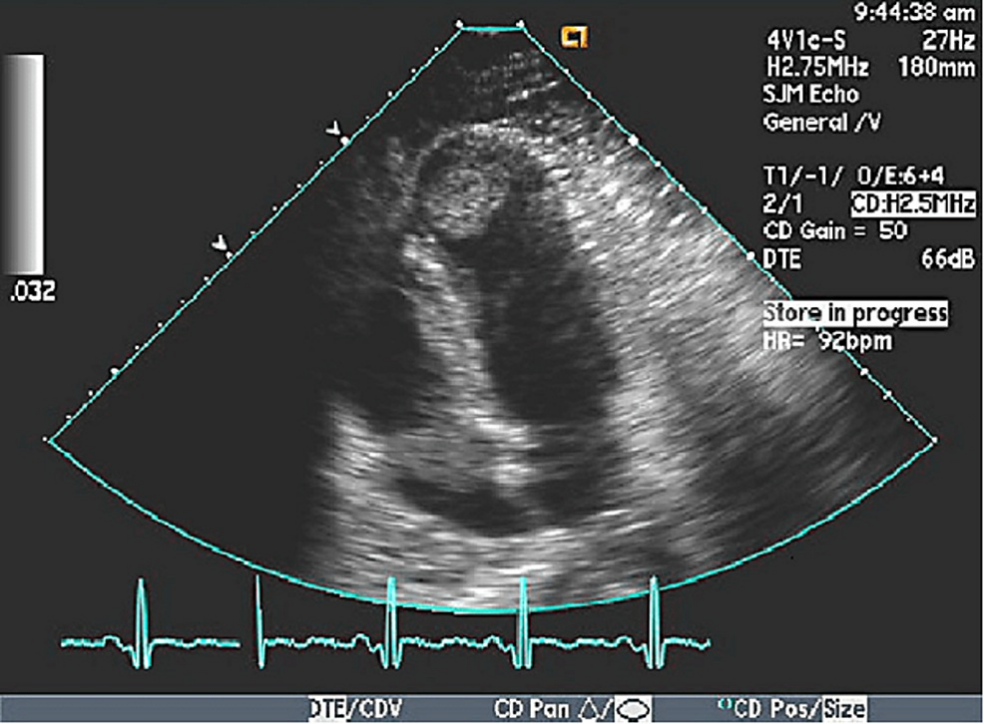
Left ventricular thrombus near the apex

The patient was started on a heparin drip to prevent the extension of the thrombus and reduce the risk of further embolization. The goal-directed medical therapy for HFrEF. He had no evidence of thrombophilia, and cardiology was consulted. They recommended transitioning the heparin drip to the direct oral anticoagulant (DOAC) rivaroxaban at 20 mg once daily for at least three months. The LV thrombus emboli were suspected of causing an ischemic stroke (Figure 2). The patient had a normal lipid profile and diabetic panel. He was counseled on abstinence from abuse, social work was consulted, and he was referred to follow-up with outpatient addiction medicine. Given his history of ischemic stroke and cardiomyopathy, he was referred to follow up with the cardiologist. The patient's symptoms improved over the next few days; he had no slurred speech or left-sided weakness. The NIHSS score was zero, and he was discharged on day 5 with oral medications.

He was seen a week later at the transitional care clinic (TCC). The patient was compliant with his medications and had no adverse effects. He had scheduled his visit for an ECHO in his cardiologist’s office. He reported abstinence from methamphetamine use. He had no recurrent symptoms. He was advised to continue his medications and follow-up visits as prescribed.

## Discussion

Methamphetamine is one of the most toxic street drugs, producing serious complications systemically affecting multiple organs. It can cause direct (myocardial injury and inflammation) and indirect (exaggerated sympathetic drive) cardiac injury [1,2]. The severe systolic dysfunction in these patients is further complicated by cardiac emboli and stroke if not detected early [3]. Methamphetamine being a sympathomimetic amine, the high catecholamine levels cause tachycardia, hypertension, vasoconstriction/spasm of the blood vessels, and possible death of the cardiac muscle [3]. Chronically this leads to fibrous tissue formation and increased heart muscle cell size [3]. Risk factors for thrombus formation include low ejection fraction, wall motion abnormalities, formation of ventricular aneurysms, and anterior MI [4]. The prevalence of left and right ventricular thrombus in methamphetamine-associated cardiomyopathy (MACM) patients is approximately 33% and 3.3%, respectively [5]. Biventricular thrombi have been a rare complication of cardiomyopathy, and only a few cases have been reported in the literature.

This patient with a six-month history of methamphetamine abuse developed heart failure with a severely reduced ejection fraction of 20-25%. The catecholamine storm resulting from methamphetamine intoxication caused the MI, leading to severe dyskinesia that led to stasis of blood and eventual thrombus formation, which was complicated by an embolic event leading to stroke. Therefore, it is crucial to consider cardioembolic events in patients with a history of methamphetamine use. Additionally, patients without any neurological signs or symptoms may be at risk of undetected intracardiac thrombi. Therefore, we strongly recommend maintaining a vigilant approach by incorporating regular surveillance echocardiography into the management plan. The two-dimensional transthoracic echocardiogram (TTE) is cost-effective, easily accessible, and has excellent specificity (85-90%) and sensitivity (95%) in detecting LV thrombus [6]. Cardiac magnetic resonance imaging (CMR) with contrast delayed enhancement (DE) has significantly better accuracy (99% specificity, 88% sensitivity) than TTE for the diagnosis of LV thrombus and is the gold standard test [6,7]. CMR can also identify fibrosis, predicting the reversibility of cardiac function [8]. Point-of-care ultrasound (POCUS) can also play a role in the detection of ventricular thrombus, particularly in acute or critical care settings where immediate bedside imaging is required. While POCUS may not offer the same level of detail, it can provide valuable information.

Our case emphasizes the need for surveillance ECHO for early detection of thrombotic complications in patients with methamphetamine-induced cardiomyopathy. Furthermore, the potential role of POCUS as an initial assessment tool and CMR as a better diagnostic cum prognostic tool can be considered [9]. When choosing these appropriate modalities, it is equally important to consider factors such as availability, expertise in interpreting the results, and patient-specific considerations.

The optimal treatment approach for such a complex condition still lacks consensus and requires further investigation. Existing studies have explored the use of DOAC compared to warfarin for LV thrombi, but more research is required in this area. Some recent case reports highlighted the use of warfarin for treating LV thrombus in patients with MACM [10,11]. The American College of Cardiology (ACC) recommends warfarin for at least three months, with repeat imaging to confirm thrombus resolution [12]. Conversely, a recent pooled analysis of randomized control trials found similar outcomes between DOACs and warfarin for LV thrombus treatment [13].

Given the limited availability of specific studies on medical therapies for MACM, it is crucial to prioritize guideline-directed medical therapy for HFrEF. In addition, prophylactic anticoagulation should be carefully evaluated individually for patients with reduced ejection fraction due to the elevated risk of LV thrombi. However, the most critical factor in promoting the potential recovery of cardiac function is the cessation of methamphetamine use. Abstinence from methamphetamines plays a vital role in optimizing the chances of cardiac function improvement.

## Conclusions

The widespread availability and affordability of methamphetamine on the streets of America have contributed to its status as a significant public health issue. The avenues for developing substance use disorders have expanded exponentially in this digital era. Clinicians need to be mindful of the potentially devastating complications of these substances - especially methamphetamine leading to complicated cardioembolic events in patients with a decreased ejection fraction. Emphasis must be laid on surveillance ECHO in MACM. Further studies are required to determine the optimal duration and intensity of anticoagulation therapy for patients with biventricular thrombi and the long-term outcomes and prognosis of MACM.
